# Iris metastasis as the initial presentation of upper gastrointestinal tract carcinoma: a case report

**DOI:** 10.1186/s13256-019-2303-5

**Published:** 2019-12-13

**Authors:** M. A. Rehman Siddiqui, Syed Zohaib Maroof Hussain, Muhammed Mubarak

**Affiliations:** 1 Section of Ophthalmology, Department of Surgery, Aga Khan University Hospital, Stadium Road, Karachi, 74800 Pakistan; 2 Department of Ophthalmology, Shahzad Eye Hospital, Karachi, Pakistan; 3 Department of Ophthalmology, South City Hospital, Karachi, Pakistan; 4 Javed I. Kazi Department of Histopathology, Sindh Institute of Urology and Transplantation (SIUT), Karachi, Pakistan

**Keywords:** Signet ring cells, Iris metastasis, Upper gastrointestinal tract tumor

## Abstract

**Background:**

We report a case of a patient with iris metastasis as the initial manifestation of a systemic cancer: upper gastrointestinal tract carcinoma.

**Case presentation:**

A 24-year-old Asian man presented to our hospital with complaints of red left eye, decreased visual acuity, pain, and photophobia for about 3 weeks with no prior history of cancer or any other medical abnormality. Ocular examination showed a pinkish white lesion on the superonasal part of the iris. The patient’s intraocular pressure was progressively increasing despite medications, followed by lymphadenopathy 4 weeks later. Comprehensive examination was performed along with a complete systemic workup, which detected systemic malignancy. Histopathology and immunohistochemistry revealed signet ring cells, which indicated an upper gastrointestinal tract tumor as a primary source of iris metastasis. The systemic condition of the patient deteriorated rapidly thereafter and led to his death in the 12th week of the disease.

**Conclusion:**

A red eye with iris lesions in otherwise healthy individuals should be considered as a possible initial manifestation of underlying systemic malignancy. Prompt referral of such patients to an oncologist is warranted.

## Introduction

Ocular metastasis is seen in 4% of systemic carcinomas. The breast and lung are common primary sites (47% and 21%, respectively). Only 4% of ocular metastases originate from the gastrointestinal tract [1, 2]. The choroid is the most common site of ocular metastasis [3]. Spread of cancer to the iris is extremely rare. Iris metastasis may present as stromal nodules or ill-defined iris thickening. It presents with atypical features such as pain, red eye, iridocyclitis, and hyphema [4, 5]. Iris metastasis as an initial manifestation of gastrointestinal tract carcinoma is even rarer. Only a few cases of iris metastasis from upper gastrointestinal tract carcinoma have been reported.

To the best of our knowledge, only one case report of iris metastasis as an initial presentation from upper gastrointestinal tract tumor has been published [6].

In the present report, we describe a case of young man with red eye and an iris lesion who was subsequently diagnosed with upper gastrointestinal tract carcinoma. The aim of this case report is to highlight the importance of systemic examination and complete workup in patients with red eye and vitiligo.

## Case presentation

A 24-year-old Asian man presented to our hospital with complaints of redness, blurring of vision, pain, and photophobia in the left eye for 3 weeks. The patient was being treated for “pink eye,” but his condition did not improve. He was generally fit and well**.** His family history was nonsignificant.

**Fig. 1:**
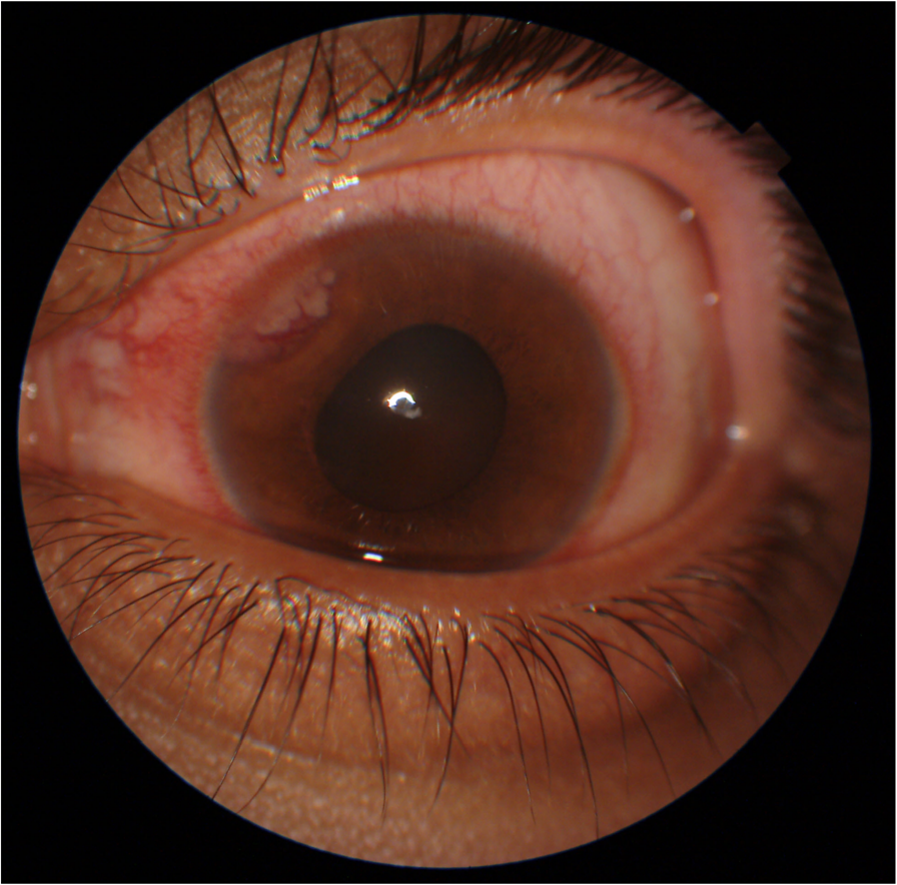
Pinkish white lesion on superonasal part of iris

**Fig. 2:**
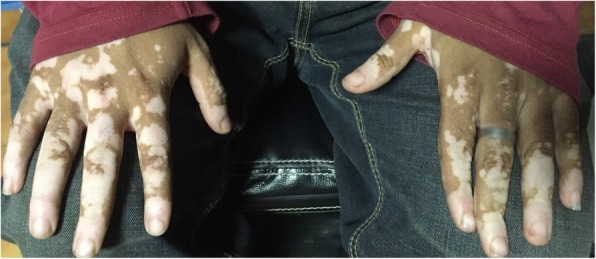
Vitiligo on both hands

**Fig. 3:**
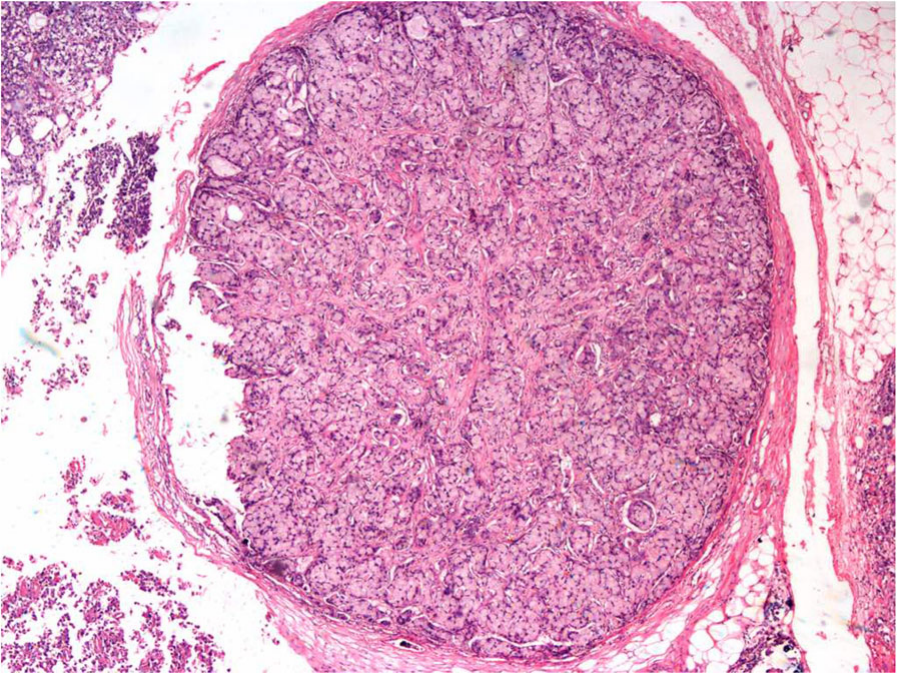
Low-power photomicrograph showing a tumor nodule embedded in surrounding fibrous adipose tissue. The tumor nodule is surrounded by a compressed pseudocapsule. No native lymphoid tissue is identified (Hematoxylin and eosin (H&E) stain; original magnification, × 40)

**Fig. 4:**
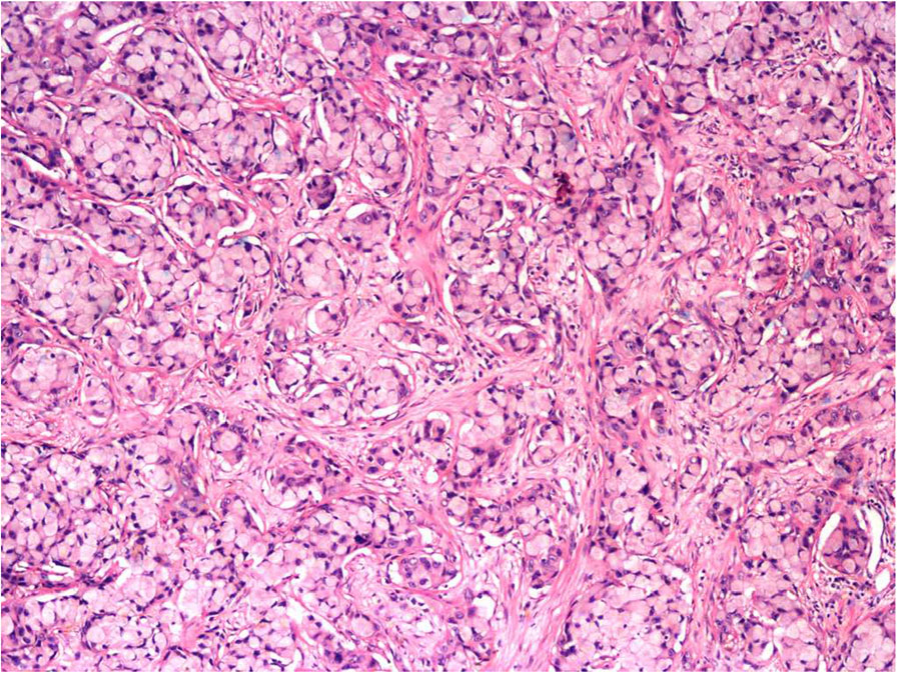
High-power view showing nests of signet ring tumor cells surrounded by delicate fibrovascular septae. The tumor cells have abundant intracellular mucin and peripherally located hyperchromatic nuclei (Hematoxylin and eosin (H&E) stain; original magnification, × 400). *Note*: Histopathological samples are available from Prof. Dr. Muhammad Mubarak at Sindh Institute of Urology and Transplantation

Ocular examination revealed a normal right eye; however, a best corrected vision of 20/30 in the left eye accompanied by circumcorneal injection, cells 2+ in the anterior chamber, and vitreous cells. His intraocular pressure (IOP) was 16 mmHg at his first visit, and the angle was open in gonioscopy. Furthermore, a pinkish white lesion on the superonasal part of the iris was observed (Fig. 1). The patient was advised to have follow-up with uveitis workup and baseline investigations; meanwhile, topical dexamethasone and cycloplegic drops were prescribed. Of note, our patient had developed vitiligo 6 months prior to the presentation (Fig. 2). At subsequent visits, vision of his left eye dropped persistently along with constant increase in IOP from 16 mmHg to 33 mmHg in just 3 weeks. For the patient’s raised IOP, topical and systemic hypotensive agents were commenced. Uveitis workup was done, and most of the test results were normal. Four weeks after presentation, he developed right-sided supraclavicular lymphadenopathy, which raised the suspicion of lymphoma. He also developed rubeosis, for which an anti-vascular endothelial growth factor injection was given. Anterior chamber paracentesis was done, which confirmed the iris lesion as a solid mass. The anterior chamber tap was reported as acellular. The results of radiology, including ultrasound of the abdomen as well as magnetic resonance imaging of brain and orbits with contrast, were normal. Computed tomography of the chest with contrast showed multiple enlarged enhancing lymph nodes with necrotic center in the cervical, mediastinal, and para-aortic regions. Some lymph nodes were extending into the apex of the right lung. Pretracheal lymph nodes were enlarged as well. Testicular tumor markers β-human chorionic gonadotropin and α-fetoprotein were normal. Tumor markers for lymphoma, namely terminal deoxynucleotidyl transferase, cyclin D1, Mum1, CD10, BCL-6, BCL-2, CK-20, villin, and CDX2, were all negative. The bone marrow biopsy report indicated 40–45% hypocellularity. Microscopic examination revealed infiltration of the iris by a malignant tumor composed of sheets and nests of signet ring cells with hyperchromatic nuclei (Figs. 3 and 4). Immunohistochemistry showed positivity of cytokeratin 7 and MUC5, raising the possibility of metastasis from the primary site in the upper gastrointestinal tract. Therefore, a final diagnosis of iris metastasis secondary to upper gastrointestinal carcinoma was made. Due to the rapidly deteriorating condition of the patient, further investigations could not be performed.

The patient was then referred and seen by an oncologist. He received intravenous chemotherapy along with high-dose corticosteroids. From week 8 to week 12, he also developed massive pleural effusion resulting in shortness of breath. He had multiple admissions to the intensive care unit during this period; however, his condition deteriorated further and led to his death in the 12th week of the disease.

## Discussion

Ocular metastasis from systemic carcinomas most commonly occurs in the choroid; involvement of the iris is less common [2]. One study reported that only 40 of 512 patients with uveal metastasis were found to have involvement of the iris, the primary sites being breast carcinoma in 16 cases, lung carcinoma in 11, carcinoid tumor in 3, melanoma in 3, colonic carcinoma in 2, and no traces of primary cancer in another 13 cases [7]. Another report, published in 2015, included 160 cases of iris metastatic tumors in 107 eyes of 104 patients, and none of the cases had the gastrointestinal tract as a primary origin of cancer [8].

In terms of diagnosis, iris tumors are sometimes misdiagnosed as inflammatory uveitis or iritis [6]. It is difficult to distinguish the origin of iris tumors. Diagnostic tools used for metastatic iris tumors include clinical findings, slit-lamp biomicroscopy, and history of systemic cancer [7]. According to Shields *et al.*, observation of typical tumors by slit-lamp biomicroscopy is the best method to diagnose iris metastasis in patients with prior history of cancer [7, 8]. In case of uncertainty, fine-needle aspiration (FNA) of the iris mass has a confirmatory role in reaching the diagnosis [7]. However, we were unable to perform FNA due to our patient’s condition, though we did perform supraclavicular lymph node biopsy and immunohistochemistry.

Treatment options include chemotherapy alone, but sometimes a combination of chemotherapy and radiotherapy is also considered, depending on tumor advancement or the patient’s response [7, 8]. Prompt treatment with chemotherapy, which is accompanied by irradiation if needed, helps to achieve better visual outcome, but the systemic prognosis is usually poor [7, 8]. Median survival from the time of diagnosis for these patients is approximately 10–13 months [7, 8].

Only a few cases of iris metastasis from upper gastrointestinal tract carcinoma were reported [2, 6, 9]. However, the primary tumor was diagnosed in majority of these cases before the presentation of iris metastasis, unlike in our case. Only one published case report of iris metastasis as a first sign of upper gastrointestinal tract carcinoma exists. That report described a case of a 75-year-old woman who presented with an iris tumor that was later identified to have metastasized from gastric signet ring cell adenocarcinoma [6].

Iris metastasis as the initial presentation of underlying undiagnosed upper gastrointestinal tract carcinoma in an otherwise healthy 24-year-old man who had developed late-onset vitiligo makes our present case report rather distinctive.

Vitiligo is an autoimmune condition of unknown etiology that causes depigmentation of different parts of the skin. Several studies have been conducted to find the relationship between autoimmune diseases and oncogenesis [10]. In one report, a 46-year-old man who was admitted to the hospital with complaints of epigastric pain and weight loss was found, on examination, to have vitiligo on both the hands and forearms. Later, the patient was diagnosed with gastric carcinoma, and surgery was performed [11]. Although vitiligo predominates in a large number of people, it is not yet confirmed that the coexistence of vitiligo and tumors are merely a coincidence or whether they have a common biological explanation [12].

## Conclusion

Iris is a rare site for secondary metastasis. Patients presenting with red eyes should receive a thorough assessment, particularly detailed evaluation of the iris, to rule out rare presentations of serious conditions such as masquerade syndromes.

## Data Availability

All the supporting data is included in the text.

## Abbreviations

FNA: Fine-needle aspiration
IOP: Intraocular pressure
